# Brief Research Report Regional Difference in TRAF2 and TRAF3 Gene Mutations in Colon Cancers

**DOI:** 10.3389/pore.2021.625438

**Published:** 2021-04-14

**Authors:** Seong Won Moon, Hyun Ji Son, Eun Ji Choi, Nam Jin Yoo, Sug Hyung Lee

**Affiliations:** ^1^ Departments of Pathology and Cancer Research Institute, Seoul, Korea; ^2^ College of Medicine, The Catholic University of Korea, Seoul, Korea

**Keywords:** TRAF2, TRAF3, mutation, cancer, expression

## Abstract

*TRAF2* and *TRAF3* genes of tumor necrosis factor receptor (TNF-R)-associated factor (TRAF) family are involved in diverse cell signaling, and function as both tumor suppressor gene and oncogene. Alterations of *TRAF2* and *TRAF3* in colon cancer (CC) along with their regional difference and microsatellite instability (MSI) are largely unknown. In the present study, we analyzed *TRAF2* and *TRAF3* frameshift mutations in 168 sporadic CCs (100 high MSI (MSI-H) and 68 microsatellite-stable (MSS) CCs). We identified *TRAF2* and *TRAF3* frameshift mutations in 4 (4%) and 3 CCs (3%) with MSI-H, respectively, but none in 68 cases of MSS CCs. Of the 168 CCs, we analyzed the mutations in multi-regions for 39 CCs (16 MSI-H and 23 MSS CCs), and discovered that 12.5% (2/16) and 6.3% (1/16) of MSI-H CCs exhibited regional difference in *TRAF2* and *TRAF3* mutations, respectively. In the multi-region samples of 23 MSS CCs, neither *TRAF2* nor *TRAF3* frameshift mutation was found. In 40% of CCs, both TRAF2 and TRAF3 expressions were increased compared to normal colon cells. Our data indicate that *TRAF2* and *TRAF3* frameshift mutations and their regional difference as well as altered expressions are present in MSI-H CCs, which could contribute to MSI-H cancer development.

## Introduction

There are six mammalian tumor necrosis factor receptor (TNF-R)-associated factor (TRAF) family proteins that possess approximately 150 amino acid TRAF domains. TRAFs behave as adaptor proteins for many receptors, including TNF-R, NOD-like receptors, CD40 and toll like receptor (TLR) that mediate interactions between the receptors and downstream effector molecules, including IRAKs, RIP1, RIP2, TAK1, MEKK1 and ASK1 [1–5]. TRAFs (TRAF2, 3, 5 and 6) also work as E3 ubiquitin ligases to regulate downstream signaling [4]. TRAF signaling pathways regulate nuclear factor-κBs (NF-κBs), mitogen-activated protein kinases, and interferon-regulatory factor pathways [1, 2]. Therefore, TRAFs function as both adaptor protein and E3 ubiquitin ligase to regulate receptor signaling in immune responses as well as other biological processes, including immune response, cell death and survival, development, and thrombosis [4]. Accordingly, TRAFs are known to be involved in the pathogenesis of many human diseases, including cancers [1, 2, 5].

Both TRAF2 and TRAF3 are closely related to each other in structure and function, and have a ring domain, a zinc finger domain, a TRAF-N domain, and a TRAF-C domain in common [4]. They play diverse roles in cell type- or context-dependent manner such as cell differentiation, survival and apoptosis via the receptor, E3 ubiquitin ligase and NF-κB activation [1, 2, 4]. They are ubiquitously expressed in various cell types, including inflammatory and epithelial cells [5]. They are involved in inflammatory and immunologic processes [5]. In cancers, TRAF2 and TRAF3 expressions are different depending on cancer types [5, 6]. In B-cell malignancies, both *TRAF2* and *TRAF3* are frequently inactivated by deletion and inactivating mutations [6]. In carcinomas, however, alterations of TRAF2 and TRAF3 are not well studied compared to hematologic malignancies [5, 6]. TRAF3 expression in pancreatic cancer is decreased compared to normal tissues [7]. Colon cancer (CC) cell lines show TRAF2 and TRAF3 expressions in 30–60% of the cases, while they are not or weakly expressed in normal colon tissues, [8]. Cancer-related roles of genes could be categorized into either oncogene or tumor suppressor gene (TSG). However, many genes show both TSG and oncogene activities, which is frequently different depending on cell or tissue contexts. As described above, TRAF2 and TRAF3 reveal both activities (i.e., cell death and survival) and thus finding gene alteration status in a specific cancer type is important for the understanding cancer-related functions of TRAF2 and TRAF3.

DNA mismatch repair (MMR) is a biological mechanism for correcting errors in nucleotide bases, alterations of which would result in microsatellite instability (MSI) and mutator phenotypes [9]. The mutator phenotype is characterized by mutation accumulation in repetitive DNA sequences (frequently mononucleotide repeats). In coding sequences, the MSI can produce frameshift mutations within affected genes that would truncate protein synthesis [9]. Gastric, colonic and endometrial cancers are the most common cancers with high MSI (MSI-H) phenotype [9]. Mounting evidence indicates that gene targets in MSI-H cancer include a growing list of cancer genes, including *TGF-β1* gene and *BAX* gene [9]. MSI-H cancers frequently harbor frameshift mutations that would inactivate TSGs. However, it is not well known whether genes with dual TSG and oncogene properties such as *TRAF2* and *TRAF3* frequently harbor inactivating mutations in cancers with MSI-H.

## Materials and Methods

### Mutation Analysis

In this study, 168 CCs from Korean patients were analyzed (100 MSI-H and 68 microsatellite-stable (MSS) CCs) (Table 1). Research approval was obtained from the institutional review board of Catholic University of Korea (MC16SISI0111). For the evaluation of MSI, we adopted five mononucleotide repeats (BAT25, BAT26, NR-21, NR-24 and MONO-27) that were known to be frequently mutated in MSI-H cancers [20]. Of the 168 CCs, multi-region sampling per CC was done in 39 CCs (16 MSI-H and 23 MSS CCs), while single region sampling was done in 129 CCs (84 MSI-H and 45 MSS CCs). In the formalin-fixed and paraffin embedded (FFPE) tissues, cancer and normal cells were separately collected as described in our earlier studies [10, 11]. The tumor cell fraction was estimated to be approximately 70–80% under microscope by a pathologist. The microdissected cell number in each case was similar among the cancers. Because we used genomic DNA from microdissected samples by a pathologist, the estimate was presumed to be reliable. There is one C7 (exon 1; primers 5′-GTA​ACG​TGC​TGT​GTG​TTC​TTC​C-3′, 5′-TAC​TTG​GCT​TCC​AGC​TTG​GTC-3′) in *TRAF2* gene, and one A7 (exon 8; primers 5′-TTG​TTA​ATT​AAT​ATG​AAA​ACC-3′, 5′-AAG​CTA​CAT​ATC​TGA​TTG​TG-3′) in *TRAF3* gene. The sequences encompassing the repeats were amplified by polymerase chain reaction (PCR) that were subsequently analyzed by single strand conformation polymorphism (SSCP) and direct DNA sequencing as described previously [10, 11]. Cancer DNA with mobility shifts in the SSCP was subsequently sequenced by Sanger DNA sequencing of both forward and reverse strands to confirm the mutated sequences (3730 DNA Analyzer, Applied Biosystem, Carlsbad, CA, United States). We analyzed regional difference in *TRAF2* and *TRAF3* frameshift mutations using 39 CCs (16 MSI-H and 23 MSS CCs) with 4–7 different areas per CC (total 92 areas).

**TABLE 1:** Summary of pathologic features of colon cancers.

Feature	MSI-H	MSS
Total cases	100	68
Mismatch repair gene expression		
MLH1 (−)	83	0
MSH2 (−)	12	0
MLH1 (+), MSH2 (+)	5	68
TNM stage		
I	18	6
II	43	33
III	35	26
IV	4	3
Location		
Cecum	20	0
Ascending colon	66	5
Transverse colon	10	5
Descending and sigmoid colon	4	25
Rectum	0	33

TNM: tumor, lymph node, metastasis, MSI-H: high microsatellite instability, MSS: stable microsatellite instability. TNM stage is defined by AJCC 8th edition.

### Immunohistochemistry

For the expression, we analyzed both TRAF2 and TRAF3 protein expressions in the CCs by immunohistochemistry with anti-TRAF2 antibody (Atlas Antibodies, Stockholm, Sweden; dilution 1/50) and anti-TRAF3 antibody (LSBio, Seattle, WA, United States; dilution 1/200), respectively. The immunohistochemistry procedures have been described in our earlier studies [11]. Briefly, sections from FFPE tissues of CCs were studied using ImmPRESS System (Vector Laboratories, Burlingame, CA, United States). We used diaminobenzidine (brown) as chromogen for the immunohistochemistry reactions and counterstained with hematoxylin (blue). Negative control of the immunostaining was the replacement of primary antibody with the blocking reagent.

## Results

**FIGURE 1:**
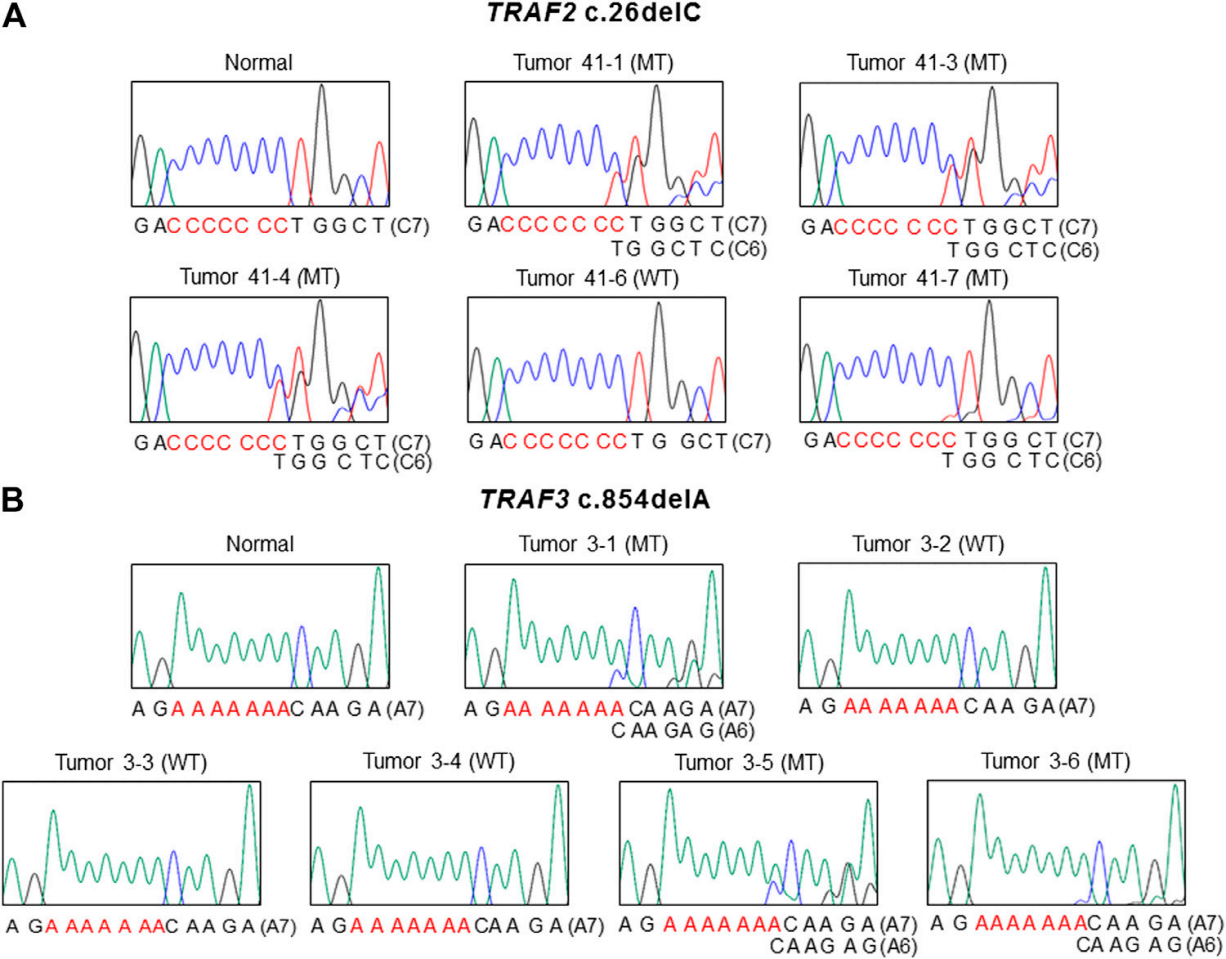
Regional difference in *TRAF2* and *TRAF3* frameshift mutation in colon cancers. Sanger DNA sequencing shows regional difference in *TRAF2* c.26delC **(A)** and *TRAF3* c.854dupA **(B)** (mutant (MT) and wild-type (WT)).

The mutation study detected somatic frameshift mutations of *TRAF2* in 4 CCs and *TRAF3* in 3 CCs by DNA sequencing (Supplementary Figure S1). They were found in CCs with MSI-H (7/100: 7.0%), but not in CCs with MSS (0/68). These mutations were either deletion or duplication of one base resulting in frameshift mutants of *TRAF2* (c.26delC (p.Pro9LeufsX77) in 4 CCs) and *TRAF3* (c.854delA (p.Asn285ThrfsX38) in 2 CCs and c.854dupA (p.Asn285LysfsX13) in one CC). We analyzed regional difference in the *TRAF2* and *TRAF3* frameshift mutations in 39 CCs (16 MSI-H and 23 MSS) (4-7 areas per CC) and found that 2 of the 16 MSI-H CCs (12.5%) exhibited different *TRAF2* mutations (3 wild and 1 mutation areas for c.26delC in one CC, 1 wild and 4 mutation areas for c.26delC in the other). The regional difference in *TRAF3* mutation was identified in one of the 16 MSI-H CCs (6.3%) (3 wild and 3 mutation areas for c.854delA) (Figure 1). We also analyzed the regional difference in the *TRAF2* and *TRAF3* mutations in 92 regions of 16 CCs by Sanger sequencing as well as by SSCP, and no additional mutation was detected by the Sanger sequencing. There was neither *TRAF2* nor *TRAF3* mutation in any of the multi-regions of 23 MSS CCs. We examined histologic difference between the regions, but did not find any discernable histologic changes. We analyzed the clinicopathologic parameters of the CCs, but there was no significant difference in them including patients’ survival between the mutated and non-mutated cases nor the regionally different and non-different cases (*p* > 0.05).

**FIGURE 2:**
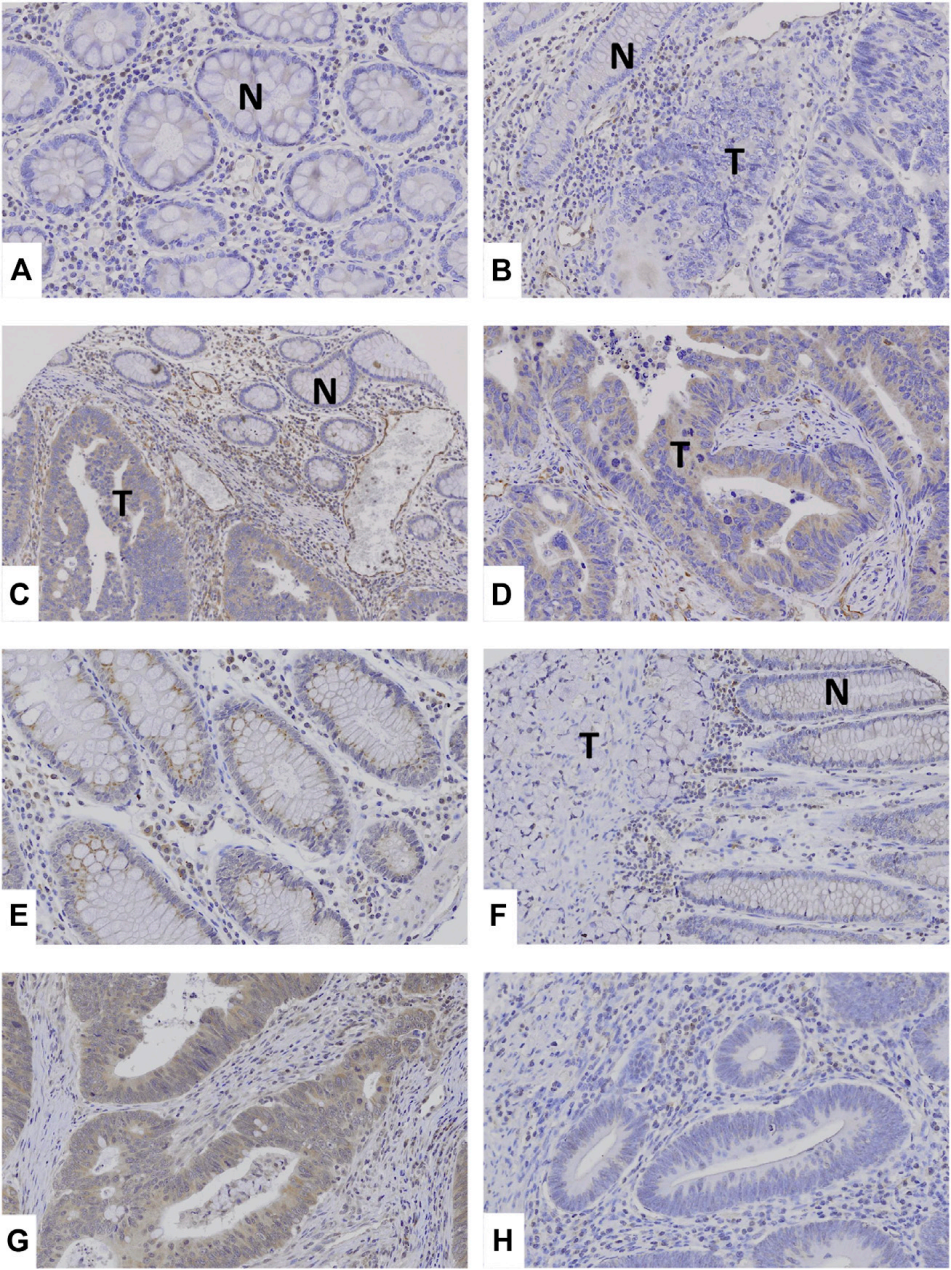
TRAF2 and TRAF3 expressions in colon cancers. **(A**–**C**) Normal colonic mucosal epithelial cells (N) show negative TRAF2 immunostaining. **(B)**: Colon cancer (T) and normal (N) cells show negative TRAF2 immunostaining. **(C,D)**: In colon cancers, the cancer cells (T) show positive TRAF2 immunostaining, which is compared to negative TRAF2 immunostaining in normal cells (N). **(E)** Normal colonic mucosal epithelial cells show weakly positive TRAF3 immunostaining. **(F)** Colon cancer (T) shows negative TRAF3 immunostaining while non-tumor mucosal cells are weakly positive (N). **(G)** Colon cancer cells show positive TRAF3 immunostaining. **(H)** Colon cancer cells show negative TRAF3.

By immunohistochemistry, we analyzed TRAF2 and TRAF3 expressions in the CCs. Normal colon mucosal cells showed negative TRAF2 and weak TRAF3 expression. In the cancers, MSS (41%, 28/68 CCs) and MSI-H (39%, 39/100 CCs) CCs showed positive TRAF2 immunostaining in cancer cells. TRAF3 was expressed MSS (46%, 31/68 CCs) and MSI-H (43%, 43/100 CCs) CCs (Figure 2). However, neither TRAF2 nor TRAF3 expressions was significantly different between MSI-H and MSS (Fisher’s exact test, *p* > 0.05). In particular, 4 CCs with the *TRAF2* and 3 CCs with *TRAF3* frameshift mutations showed negative TRAF2 and TRAF3 expressions, respectively.

## Discussion

In this study, we attempt to address following questions whether: 1) CCs harbor *TRAF2* and *TRAF3* frameshift mutations in MSI-H-specific manner: 2) there is any regional difference in *TRAF2* and *TRAF3* frameshift mutations and 3) there are any expressional alterations of TRAF2 and TRAF3 in CCs. We discovered somatic frameshift mutations in MSI-H CCs that would produce premature stops in TRAF2 and TRAF3 proteins. Furthermore, regional difference in the frameshift mutations of *TRAF2* and *TRAF3* was not uncommon in the MSI-H CCs (3/16). Finally, we identified that TRAF2 and TRAF3 protein expressions were increased in approximately 40% of CCs irrespective of the MSH status compared to those in normal colon cells, which is consistent with earlier cell line studies [8].

In this study, we identified *TRAF2* and *TRAF3* mutations in 4 (4%) and 3 (3%) CCs with MSI-H, respectively. For these, we searched the frameshift mutations in other mutation repositories and compared their prevalence. In 190 MSI-H cancers with altered Bethesda MSI markers (48 CCs, 64 gastric cancers and 75 uterine endometrial cancers) [12], there are 6 *TRAF2* mutations (5 gastric, 1 endometrial and 0 colon cancers; 3.2%) and 6 *TRAF3* mutations (2 gastric, 2 endometrial and 2 colon cancers; 3.2%) with insertion or deletion in the 190 MSI-H cancers. There is significant difference neither in *TRAF2* nor *TRAF3* mutation between our and other data (*p* > 0.05). As for CCs, there is significant difference neither in *TRAF2* nor *TRAF3* mutation between our and other data (*p* > 0.05).

The TRAF2 consists of 501 amino acids, which contains a ring domain (amino acids 26–97), a zinc finger domain (100–249), a TRAF-N domain (272–355), and a TRAF-C domain (356–501) [5, 13]. The frameshift mutations detected in the present study would result in premature stops of amino acid synthesis in TRAF2 and TRAF3 proteins and hence resemble a typical loss-of-function mutation [9]. The *TRAF2* mutation (p.Pro9LeufsX77) identified in the present study would result in production of only a short protein with 8 amino acids without any of the domains, which might lose the activities or be degraded by nonsense-mediated mRNA decay that is frequently present in MSI-H cancers [14]. The TRAF3 is 568 amino acids long with a ring domain (amino acids 46–107), a zinc finger domain (111–265), a TRAF-N domain (288–419), and a TRAF-C domain (420–568) [5, 13]. Both of the *TRAF3* mutations (p.Asn285ThrfsX38 and p.Asn285LysfsX13) identified in the present study would result in production of the ring domain and the zinc finger domain without the TRAF-N and TRAF-C domains [3], which might lose the activities or be degraded by nonsense-mediated mRNA decay that is frequently present in MSI-H cancers [14]. Both TRAF-N and TRAF-C domains of TRAF3 are important for CD40 binding and homodimerization [15]. The TRAF3 mutant devoid of TRAF-N and TRAF-C domains does not activate NF-κB signals [14]. The *TRAF2* and *TRAF3* mutations found in our study were heterozygous forms. Experimentally, both truncation mutants of TRAF2 and TRAF3 reveal dominant-negative effect of TNF-induced NF-κB activation [14]. In this sense, the *TRAF2* and *TRAF3* mutations might inactivate their activities, but their biological functions and contributions to cancer development are not explored in our study and should be further studied.

In primary colon cancer cells, TRAF2 mediates chemotherapy-induced apoptosis by acting in TRAF2-JNK-p53 axis [16]. TRAF2-deficient mice spontaneously develop inflammatory bowel diseases, which could be a background for developing inflammation-induced cancer [17]. NLRP12-deficient mice, a mouse model with functional relevance to TRAF3, is highly susceptible to colitis and colitis-associated colon cancer [18]. miR-32-TRAF3-mediated inhibition of NF-κB pathway protects colorectal epithelium against colon carcinogenesis [19]. These studies support TSG functions for TRAF2 and TRAF3 in colon tumorigenesis.

Under suitable conditions, SSCP is capable of detecting over 90% of mutations occurring within any sequences, and the sensitivity of PCR-SSCP is generally believed to be high if the fragments are shorter than 200 bp [20]. Because we have analyzed the samples by SSCP with products shorter than 200 bp, it can be considered that the missing of *TRAF2* and *TRAF3* mutations, if any, would be rare in this study.

The results may suggest that TSG functions of TRAF2 and TRAF3 such as cell death induction [1–5] might be inactivated by the frameshift mutations, which could contribute to cell death evasion in cancer pathogenesis. As for the oncogenic functions of these genes such as NF-κB activation and cell survival, the consequences of the inactivating mutations remain unclear in the CCs. Presence of the regional difference might further alter consequences of these mutations, too. One possible way to address the consequences would be to find any difference in pathologic and clinical parameters between mutated and non-mutated CCs or regionally different and non-different CCs. However, probably due to the small number of cases, we were not able to find such a parameter. Our study suggests that inactivating mutations of *TRAF2* and *TRAF3* as well as their regional difference might be involved in the pathogenesis of MSI-H CCs, but the significance should be further studied in a larger cohort.

## Data Availability

The original contributions presented in the study are included in the article/Supplementary Material, further inquiries can be directed to the corresponding author.
