# Supplementary figures and images for: Brief Research Report Regional Difference in TRAF2 and TRAF3 Gene Mutations in Colon Cancers
Source: Pathol Oncol Res. 2021 Apr 14;27:625438. doi: 10.3389/pore.2021.625438 (PMC8262244; doi:10.3389/pore.2021.625438)

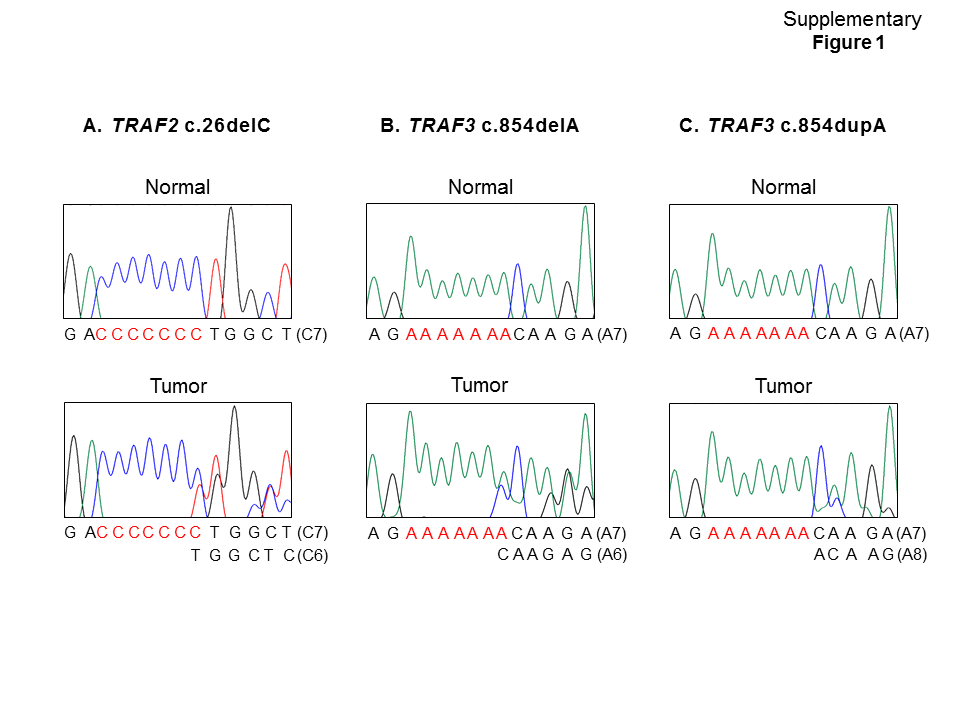

Supplement: Supplementary file 1 [file Image1.TIF]
